# Ethyl 3-bromo-1-(3-chloro­pyridin-2-yl)-1*H*-pyrazole-5-carboxyl­ate

**DOI:** 10.1107/S1600536808039329

**Published:** 2008-12-13

**Authors:** Hai-Bing He, Shan Liu, Hai-Jun Tan, Ming Xia, Hong-Jun Zhu

**Affiliations:** a Department of Applied Chemistry, College of Science, Nanjing University of Technology, Nanjing 210009, People’s Republic of China

## Abstract

The title compound, C_11_H_9_BrClN_3_O_2_, is an inter­mediate in the synthesis of Rynaxypyre, a new insecticidal anthranilic diamide used as a potent and selective ryanodine receptor activator. The dihedral angle between the aromatic ring planes is 78.7 (2)°.

## Related literature

For the synthetic procedure, see: Lahm *et al.* (2007 ▶). For bond-length data, see: Allen *et al.* (1987 ▶).
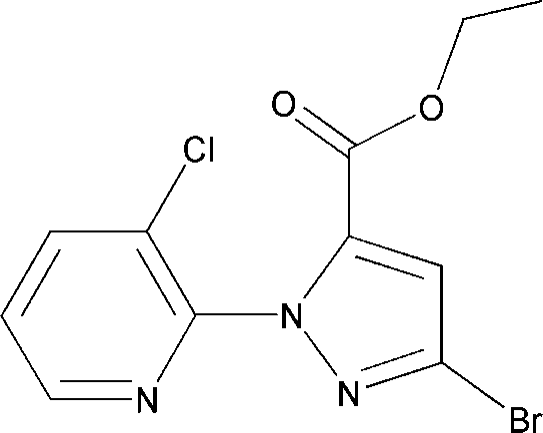


## Experimental

### 

#### Crystal data

C_11_H_9_BrClN_3_O_2_
*M* *_r_* = 330.57
Orthorhombic, 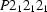

*a* = 7.404 (2) Å
*b* = 10.024 (2) Å
*c* = 17.072 (3) Å
*V* = 1267.0 (4) Å^3^
*Z* = 4
Mo *K*α radiation
μ = 3.45 mm^−1^
*T* = 298 (2) K
0.40 × 0.30 × 0.30 mm

#### Data collection

Enraf–Nonius CAD-4 diffractometer
Absorption correction: ψ scan (North *et al.*, 1968 ▶) *T*_min_ = 0.339, *T*_max_ = 0.424 (expected range = 0.284–0.355)
2295 measured reflections
1347 independent reflections
959 reflections with *I* > 2σ(*I*)
*R*_int_ = 0.051
3 standard reflections every 200 reflections intensity decay: 1%

#### Refinement

*R*[*F* ^2^ > 2σ(*F* ^2^)] = 0.056
*wR*(*F* ^2^) = 0.129
*S* = 1.00
1347 reflections
163 parameters
H-atom parameters constrained
Δρ_max_ = 0.33 e Å^−3^
Δρ_min_ = −0.42 e Å^−3^
Absolute structure: Flack (1983 ▶), 15 Friedel pairs
Flack parameter: 0.37

### 

Data collection: *CAD-4 Software* (Enraf–Nonius, 1985 ▶); cell refinement: *CAD-4 Software*; data reduction: *XCAD4* (Harms & Wocadlo, 1995 ▶); program(s) used to solve structure: *SHELXS97* (Sheldrick, 2008 ▶); program(s) used to refine structure: *SHELXL97* (Sheldrick, 2008 ▶); molecular graphics: *SHELXTL* (Sheldrick, 2008 ▶); software used to prepare material for publication: *SHELXTL*.

## Supplementary Material

Crystal structure: contains datablocks I, global. DOI: 10.1107/S1600536808039329/im2086sup1.cif

Structure factors: contains datablocks I. DOI: 10.1107/S1600536808039329/im2086Isup2.hkl

Additional supplementary materials:  crystallographic information; 3D view; checkCIF report

## Figures and Tables

**Fig. 1.**
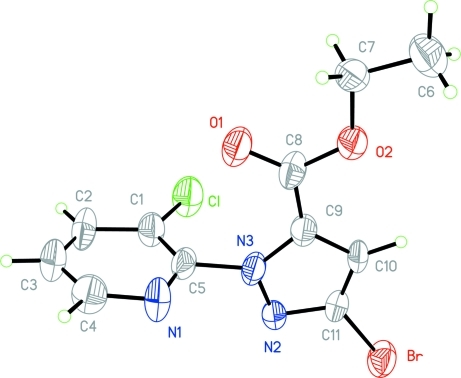
A drawing of the title molecular structure, with the atom-numbering scheme. Displacement ellipsoids are drawn at the 50% probability level.

**Fig. 2.**
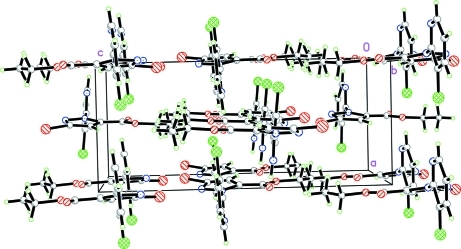
A packing diagram for (I).

## supplementary crystallographic information

### Comment

Ethyl 3-bromo-1-(3-chloropyridin-2-yl)-1*H*-pyrazole-5-carboxylate is one of the important intermediates in the synthesis of Rynaxypyre, a new insecticidal anthranilic diamide used as a potent and selective ryanodine receptor activator (Lahm *et al.*, 2007).

The molecular structure of (I) is shown in Fig. 1, a full list of geometric parameters is given in the supplementary material. The bond lengths and angles are within normal ranges (Allen *et al.*, 1987). The dihedral angle of the rings A (C1—C5/N1) and B(C9—C11/N2/N3) is measured to 78.7 (2)°.

No obvious intra- or inter-molecular hydrogen bonds were observed in the crystal structure (Fig. 2).

### Experimental

The title compound (I) was synthesized by a method reported in the literature (Lahm *et al.*, 2007). Crystals of the title compound were obtained by dissolving ethyl 3-bromo-1-(3-chloropyridin-2-yl)-1*H*-pyrazole-5-carboxylate (3.3 g, 10.0 mmol) in acetonitrile (60 ml) and evaporating the solvent slowly at room temperature for about 20 d.

### Refinement

H atoms were positioned geometrically, with O—H = 0.82 Å and C—H = 0.93Å for aromatic H, 0.97 Å for CH_2_ and 0.96 Å for CH_3_ groups. Hydrogen atoms were constrained to ride on their parent atoms, with *U*_iso_(H) = *xU*_eq_(C/O), where *x* = 1.2 for aromatic H and *x* = 1.5 for other H.

### Crystal data

**Table:**

C_11_H_9_BrClN_3_O_2_	*F*(000) = 656
*M**_r_* = 330.57	*D*_x_ = 1.733 Mg m^−^^3^
Orthorhombic, *P*2_1_2_1_2_1_	Mo *K*α radiation, λ = 0.71073 Å
Hall symbol: P 2ac 2ab	Cell parameters from 25 reflections
*a* = 7.404 (2) Å	θ = 9–13°
*b* = 10.024 (2) Å	µ = 3.45 mm^−^^1^
*c* = 17.072 (3) Å	*T* = 298 K
*V* = 1267.0 (4) Å^3^	Block, colourless
*Z* = 4	0.40 × 0.30 × 0.30 mm

### Data collection

**Table:**

Enraf–Nonius CAD-4 diffractometer	959 reflections with *I* > 2σ(*I*)
Radiation source: fine-focus sealed tube	*R*_int_ = 0.051
graphite	θ_max_ = 25.3°, θ_min_ = 2.4°
ω/2θ scans	*h* = 0→8
Absorption correction: ψ scan (North *et al.*, 1968)	*k* = 0→12
*T*_min_ = 0.339, *T*_max_ = 0.424	*l* = −20→20
2295 measured reflections	3 standard reflections every 200 reflections
1347 independent reflections	intensity decay: 1%

### Refinement

**Table:**

Refinement on *F*^2^	Secondary atom site location: difference Fourier map
Least-squares matrix: full	Hydrogen site location: inferred from neighbouring sites
*R*[*F*^2^ > 2σ(*F*^2^)] = 0.056	H-atom parameters constrained
*wR*(*F*^2^) = 0.129	*w* = 1/[σ^2^(*F*_o_^2^) + (0.063*P*)^2^ + 0.5*P*] where *P* = (*F*_o_^2^ + 2*F*_c_^2^)/3
*S* = 1.00	(Δ/σ)_max_ = 0.001
1347 reflections	Δρ_max_ = 0.33 e Å^−^^3^
163 parameters	Δρ_min_ = −0.42 e Å^−^^3^
0 restraints	Absolute structure: Flack (1983), 155 Frieldel pairs
Primary atom site location: structure-invariant direct methods	Flack parameter: 0.37

### Special details

**Table:**

Geometry. All e.s.d.'s (except the e.s.d. in the dihedral angle between two l.s. planes) are estimated using the full covariance matrix. The cell e.s.d.'s are taken into account individually in the estimation of e.s.d.'s in distances, angles and torsion angles; correlations between e.s.d.'s in cell parameters are only used when they are defined by crystal symmetry. An approximate (isotropic) treatment of cell e.s.d.'s is used for estimating e.s.d.'s involving l.s. planes.
Refinement. Refinement of *F*^2^ against ALL reflections. The weighted *R*-factor *wR* and goodness of fit *S* are based on *F*^2^, conventional *R*-factors *R* are based on *F*, with *F* set to zero for negative *F*^2^. The threshold expression of *F*^2^ > σ(*F*^2^) is used only for calculating *R*-factors(gt) *etc*. and is not relevant to the choice of reflections for refinement. *R*-factors based on *F*^2^ are statistically about twice as large as those based on *F*, and *R*- factors based on ALL data will be even larger.

### Fractional atomic coordinates and isotropic or equivalent isotropic displacement parameters (Å^2^)

**Table:**

	*x*	*y*	*z*	*U*_iso_*/*U*_eq_	
Br	0.03831 (14)	0.07498 (8)	0.79256 (5)	0.0580 (3)	
Cl	0.2975 (3)	0.5607 (2)	0.90139 (15)	0.0661 (6)	
O1	0.0029 (10)	0.4767 (5)	1.0803 (3)	0.0645 (19)	
N1	−0.2392 (8)	0.5697 (7)	0.9219 (4)	0.0481 (16)	
C1	0.0862 (11)	0.6189 (7)	0.9087 (5)	0.046 (2)	
C2	0.0441 (15)	0.7564 (7)	0.9030 (4)	0.056 (2)	
H2A	0.1355	0.8190	0.8964	0.067*	
N2	−0.0180 (10)	0.3292 (5)	0.8505 (3)	0.0457 (17)	
O2	0.0190 (8)	0.2604 (4)	1.1121 (3)	0.0483 (14)	
N3	−0.0260 (10)	0.3932 (5)	0.9196 (3)	0.0442 (16)	
C3	−0.1273 (14)	0.7949 (8)	0.9073 (6)	0.059 (2)	
H3A	−0.1564	0.8848	0.9027	0.071*	
C4	−0.2629 (11)	0.7014 (8)	0.9187 (5)	0.055 (2)	
H4A	−0.3801	0.7333	0.9245	0.066*	
C5	−0.0628 (13)	0.5354 (6)	0.9191 (4)	0.045 (2)	
C6	0.0235 (18)	0.1666 (8)	1.2393 (5)	0.074 (3)	
H6A	0.0324	0.1860	1.2943	0.111*	
H6B	0.1218	0.1099	1.2241	0.111*	
H6C	−0.0889	0.1224	1.2289	0.111*	
C7	0.0317 (13)	0.2919 (7)	1.1945 (4)	0.0476 (19)	
H7A	−0.0671	0.3499	1.2097	0.057*	
H7B	0.1444	0.3377	1.2052	0.057*	
C8	0.0085 (12)	0.3603 (7)	1.0617 (4)	0.046 (2)	
C9	0.0042 (11)	0.3108 (6)	0.9816 (4)	0.041 (2)	
C10	0.0303 (12)	0.1880 (6)	0.9503 (4)	0.0413 (17)	
H10A	0.0526	0.1092	0.9773	0.050*	
C11	0.0173 (11)	0.2032 (6)	0.8719 (4)	0.0383 (18)	

### Atomic displacement parameters (Å^2^)

**Table:**

	*U*^11^	*U*^22^	*U*^33^	*U*^12^	*U*^13^	*U*^23^
Br	0.0822 (6)	0.0377 (4)	0.0539 (4)	0.0033 (5)	0.0035 (5)	−0.0055 (4)
Cl	0.0537 (13)	0.0473 (14)	0.0974 (17)	0.0016 (12)	0.0030 (12)	0.0009 (13)
O1	0.107 (6)	0.032 (3)	0.054 (3)	−0.003 (3)	−0.006 (4)	−0.004 (2)
N1	0.047 (4)	0.028 (4)	0.070 (4)	0.004 (4)	0.019 (3)	0.005 (4)
C1	0.045 (5)	0.034 (4)	0.058 (5)	0.004 (3)	0.002 (4)	0.000 (3)
C2	0.082 (7)	0.026 (4)	0.059 (5)	0.001 (5)	0.018 (6)	0.003 (3)
N2	0.067 (5)	0.028 (3)	0.042 (3)	0.003 (3)	−0.014 (4)	0.002 (2)
O2	0.070 (4)	0.035 (3)	0.040 (2)	0.000 (3)	−0.004 (3)	−0.001 (2)
N3	0.066 (4)	0.024 (3)	0.043 (3)	−0.003 (3)	−0.001 (3)	−0.001 (2)
C3	0.071 (7)	0.030 (5)	0.077 (6)	0.009 (5)	0.004 (6)	−0.003 (4)
C4	0.034 (5)	0.052 (5)	0.079 (6)	0.001 (4)	0.002 (5)	−0.001 (5)
C5	0.073 (6)	0.025 (4)	0.036 (4)	−0.010 (4)	−0.005 (4)	0.007 (3)
C6	0.113 (9)	0.058 (5)	0.050 (5)	0.006 (6)	−0.006 (6)	0.013 (4)
C7	0.059 (5)	0.043 (4)	0.041 (4)	0.010 (4)	−0.006 (4)	−0.005 (3)
C8	0.057 (7)	0.029 (4)	0.052 (4)	−0.008 (4)	0.009 (4)	0.001 (3)
C9	0.052 (6)	0.030 (4)	0.041 (4)	−0.001 (4)	0.000 (4)	0.001 (3)
C10	0.057 (5)	0.019 (3)	0.048 (4)	0.002 (4)	−0.003 (4)	0.003 (3)
C11	0.044 (5)	0.025 (3)	0.046 (4)	−0.004 (3)	0.003 (4)	0.001 (3)

### Geometric parameters (Å, °)

**Table:**

Br—C11	1.874 (6)	N3—C5	1.452 (8)
Cl—C1	1.675 (8)	C3—C4	1.387 (12)
O1—C8	1.210 (7)	C3—H3A	0.9300
N1—C4	1.334 (9)	C4—H4A	0.9300
N1—C5	1.351 (11)	C6—C7	1.472 (9)
C1—C5	1.396 (11)	C6—H6A	0.9600
C1—C2	1.416 (10)	C6—H6B	0.9600
C2—C3	1.328 (12)	C6—H6C	0.9600
C2—H2A	0.9300	C7—H7A	0.9700
N2—C11	1.340 (8)	C7—H7B	0.9700
N2—N3	1.344 (7)	C8—C9	1.454 (10)
O2—C8	1.323 (8)	C9—C10	1.356 (9)
O2—C7	1.445 (7)	C10—C11	1.351 (9)
N3—C9	1.361 (8)	C10—H10A	0.9300
			
C4—N1—C5	112.2 (6)	H6A—C6—H6B	109.5
C5—C1—C2	114.7 (8)	C7—C6—H6C	109.5
C5—C1—Cl	122.6 (6)	H6A—C6—H6C	109.5
C2—C1—Cl	122.6 (7)	H6B—C6—H6C	109.5
C3—C2—C1	119.3 (9)	O2—C7—C6	108.5 (6)
C3—C2—H2A	120.4	O2—C7—H7A	110.0
C1—C2—H2A	120.4	C6—C7—H7A	110.0
C11—N2—N3	102.7 (5)	O2—C7—H7B	110.0
C8—O2—C7	118.2 (5)	C6—C7—H7B	110.0
N2—N3—C9	112.7 (5)	H7A—C7—H7B	108.4
N2—N3—C5	118.2 (5)	O1—C8—O2	124.1 (7)
C9—N3—C5	129.1 (5)	O1—C8—C9	125.1 (7)
C2—C3—C4	120.2 (8)	O2—C8—C9	110.8 (6)
C2—C3—H3A	119.9	C10—C9—N3	105.5 (6)
C4—C3—H3A	119.9	C10—C9—C8	132.6 (6)
N1—C4—C3	125.4 (8)	N3—C9—C8	121.9 (6)
N1—C4—H4A	117.3	C11—C10—C9	106.1 (6)
C3—C4—H4A	117.3	C11—C10—H10A	126.9
N1—C5—C1	128.0 (6)	C9—C10—H10A	126.9
N1—C5—N3	115.5 (7)	N2—C11—C10	113.0 (6)
C1—C5—N3	116.2 (8)	N2—C11—Br	117.8 (5)
C7—C6—H6A	109.5	C10—C11—Br	129.2 (5)
C7—C6—H6B	109.5		
			
C5—C1—C2—C3	−0.4 (12)	C8—O2—C7—C6	174.4 (8)
Cl—C1—C2—C3	178.2 (8)	C7—O2—C8—O1	−2.5 (13)
C11—N2—N3—C9	0.4 (9)	C7—O2—C8—C9	177.6 (7)
C11—N2—N3—C5	180.0 (7)	N2—N3—C9—C10	−0.9 (10)
C1—C2—C3—C4	1.2 (15)	C5—N3—C9—C10	179.6 (8)
C5—N1—C4—C3	4.9 (12)	N2—N3—C9—C8	177.7 (7)
C2—C3—C4—N1	−3.8 (16)	C5—N3—C9—C8	−1.8 (14)
C4—N1—C5—C1	−4.2 (11)	O1—C8—C9—C10	171.0 (10)
C4—N1—C5—N3	−177.9 (6)	O2—C8—C9—C10	−9.1 (13)
C2—C1—C5—N1	2.1 (12)	O1—C8—C9—N3	−7.1 (14)
Cl—C1—C5—N1	−176.5 (6)	O2—C8—C9—N3	172.8 (8)
C2—C1—C5—N3	175.8 (6)	N3—C9—C10—C11	0.9 (10)
Cl—C1—C5—N3	−2.8 (10)	C8—C9—C10—C11	−177.4 (9)
N2—N3—C5—N1	89.5 (9)	N3—N2—C11—C10	0.2 (10)
C9—N3—C5—N1	−91.0 (10)	N3—N2—C11—Br	179.4 (6)
N2—N3—C5—C1	−85.1 (9)	C9—C10—C11—N2	−0.7 (10)
C9—N3—C5—C1	94.4 (10)	C9—C10—C11—Br	−179.8 (6)
